# Nrf2 as a redox checkpoint in autoimmune joint inflammation: microenvironmental redox control across the arthritis spectrum

**DOI:** 10.3389/fimmu.2026.1775908

**Published:** 2026-03-11

**Authors:** Mingwang Zhou, Haiyuan Gao, Xiaoping Wang, Zhenhua Shi, Xing Yang, YuNan Li, XinHao Li, Yongqiang Zhao

**Affiliations:** 1 Department of Joint Orthopaedics, Gansu Provincial Hospital of Traditional Chinese Medicine, Lanzhou, Gansu, China; 2 Department of Joint Orthopaedics, First Affiliated Hospital of Gansu University of Traditional Chinese Medicine, Lanzhou, Gansu, China; 3 Institute of Orthopedics, Gansu Provincial Institute of Traditional Chinese Medicine, Lanzhou, Gansu, China; 4 Clinical College of Traditional Chinese Medicine, Gansu University of Traditional Chinese Medicine, Lanzhou, Gansu, China; 5 The Translational Medicine Research Center, Gansu Provincial Hospital of Traditional Chinese Medicine, Lanzhou, Gansu, China; 6 Urology, Gansu Provincial Hospital of Traditional Chinese Medicine, Lanzhou, Gansu, China

**Keywords:** arthritis, autoimmune synovitis, autoimmunity, inflammation, Nrf2, oxidative stress, reactive oxygen species

## Abstract

Arthritis comprises a spectrum of immune-mediated joint disorders, with rheumatoid arthritis (RA) representing prototypic autoimmunity and psoriatic arthritis (PsA) and ankylosing spondylitis (AS) spanning an autoinflammation–autoimmunity continuum. Across this spectrum, oxidative stress and inflammatory signaling reinforce each other within synovial/entheseal niches, sustaining immune activation and progressive structural damage. Excess reactive oxygen species (ROS) injure chondrocytes and synoviocytes, activate NF-κB and the NLRP3 inflammasome, and reprogram stromal–immune interactions; inflammatory mediators further increase ROS via NADPH oxidases, mitochondrial dysfunction, and immunometabolic perturbations, sustaining a “ROS–inflammation–ROS” loop. Nuclear factor erythroid 2–related factor 2 (Nrf2) is a redox-responsive transcription factor that, upon release from Keap1, drives antioxidant response element–dependent cytoprotective programs. Beyond antioxidation, Nrf2 can dampen NF-κB-linked transcription and modulate ferroptosis, pyroptosis, and autophagy while shaping macrophage and fibroblast-like synoviocyte states. Collectively, these actions position Nrf2 as a context-dependent redox checkpoint that may constrain inflammatory amplification and tune autoimmune-relevant processes (e.g., inflammatory antigen presentation and effector persistence) largely via microenvironmental remodeling rather than direct TCR/BCR inhibition. Here, we (i) map Nrf2-dependent versus Nrf2-independent nodes in the oxidative stress–inflammation circuit; (ii) compare cell type– and subtype-specific Nrf2 functions across RA, PsA, and AS; (iii) summarize pharmacologic and natural-product Nrf2 activators together with joint-targeted delivery strategies; and (iv) discuss evidence and gaps for Nrf2 in core autoimmune mechanisms, including self-tolerance, antigen handling, and pathogenic immune memory. This synthesis highlights Nrf2 as a mechanistic bridge between redox balance and immune regulation, informing Nrf2-centered therapies for autoimmune and immune-mediated arthritides.

## 1 Introduction

Arthritis comprises a spectrum of immune-mediated joint disorders in which breakdown of self-tolerance and dysregulated innate–adaptive immune crosstalk sustain chronic synovial inflammation and progressive tissue damage (1). Rheumatoid arthritis (RA) exemplifies classic autoimmunity, with autoreactive T and B cells, antigen presentation within the synovium, and disease-defining autoantibodies driving pathogenic autoreactive effector programs; by contrast, psoriatic arthritis (PsA) and ankylosing spondylitis (AS) often display prominent IL-23/IL-17–skewed inflammation and tissue-site–specific immune niches along an autoinflammation–autoimmunity continuum (2–4). Across this spectrum, the synovial/entheseal microenvironment integrates cytokine networks, immunometabolic stress, and regulated cell-death pathways into feed-forward circuits that amplify immune activation. Reactive oxygen species (ROS) are not merely by-products of inflammation but instructive signals that can shape antigen processing, inflammasome activity, and effector differentiation (5). Nuclear factor erythroid 2–related factor 2 (Nrf2), a master redox-responsive transcription factor, couples oxidative cues to cytoprotective and immunoregulatory programs, influencing dendritic-cell maturation, macrophage polarization, T-cell fate decisions, and stromal-cell pathogenicity (6). In this review, we synthesize how Nrf2-centered redox control intersects with core autoimmune mechanisms to remodel the joint niche and highlight Nrf2 as a mechanistic bridge—and potential therapeutic lever—to disrupt self-sustaining immune pathology across the autoimmunity-autoinflammation spectrum of inflammatory arthritides.

**Figure 1:**
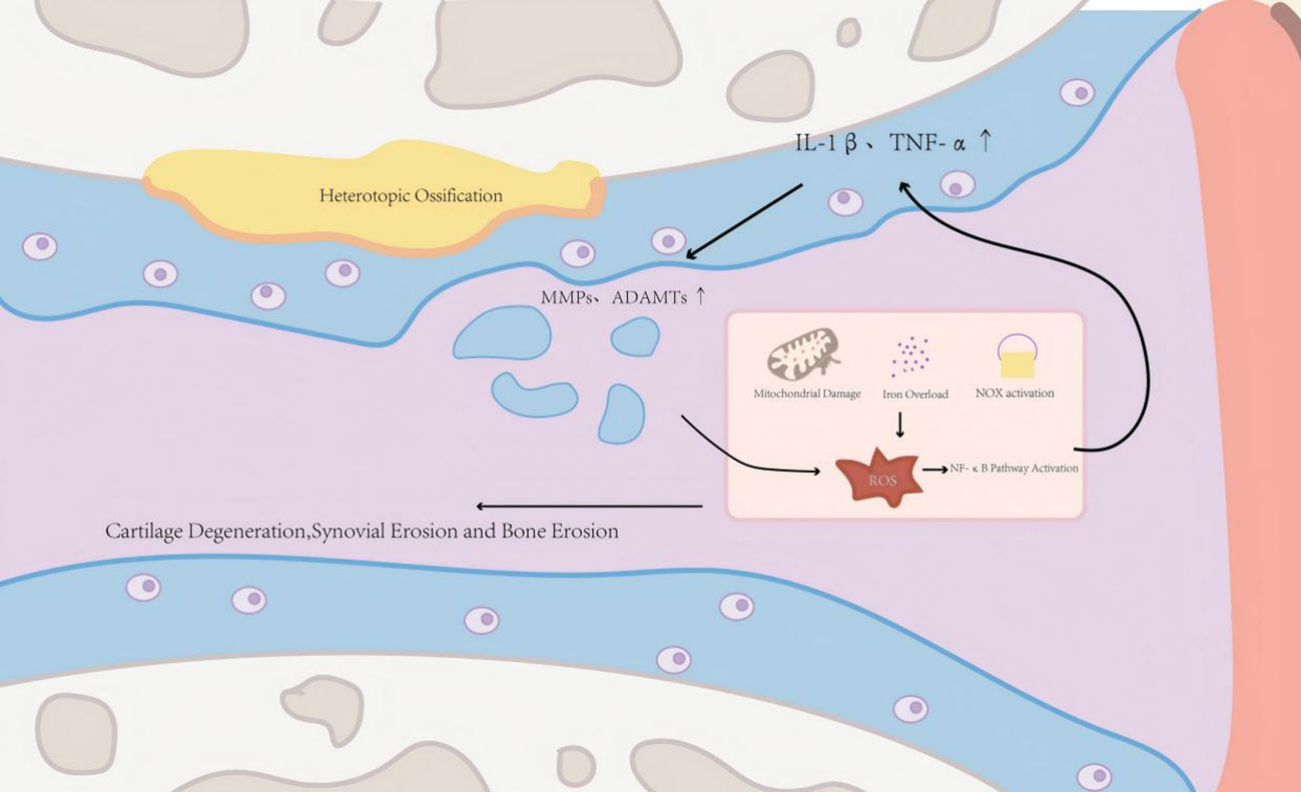
Oxidative stress–inflammation circuit in arthritis progression. ROS generated by mitochondrial dysfunction, iron overload, and NOX activation promote NF-κB signaling and pro-inflammatory cytokine production (e.g., IL-1β and TNF-α). These signals induce catabolic enzymes (MMPs/ADAMTS), resulting in extracellular matrix (ECM) degradation, cartilage/bone damage, and ectopic ossification, while inflammatory mediators further enhance ROS generation, sustaining the circuit. ROS, reactive oxygen species; NOX, NADPH oxidase; NF-κB, nuclear factor-kappa B; IL-1β, interleukin-1 beta; TNF-α, tumor necrosis factor-alpha; MMPs, matrix metalloproteinases; ADAMTs, A disintegrin and metalloproteinase with thrombospondin motifs.

A key unifying concept is that ROS and inflammatory mediators mutually reinforce each other, forming a self-amplifying circuit that accelerates tissue injury and disease progression (7). In chronic arthritic settings, persistent ROS accumulation undermines chondrocyte viability through senescence, apoptosis, and aberrant autophagy, promotes extracellular matrix breakdown (8), and activates synoviocytes and infiltrating immune cells to produce catabolic enzymes and cytokines. Conversely, pro-inflammatory cytokines and danger signals enhance NADPH oxidase activity, disrupt mitochondrial electron transport, and reshape cellular metabolism, thereby further elevating ROS generation and sustaining oxidative stress–mediated inflammatory responses (9). This reciprocal reinforcement provides a mechanistic rationale for disease-modifying strategies aimed at disrupting the ROS-inflammation circuit (**Figure 1**).

In this context, the nuclear factor erythroid 2-related factor 2 (Nrf2) pathway has attracted increasing attention as a central regulatory system coordinating antioxidant and anti-inflammatory responses (10). Under oxidative or electrophilic stress, Nrf2 dissociates from Kelch-like ECH-associated protein 1 (Keap1), translocates to the nucleus, and induces transcription of antioxidant response element (ARE)-containing genes, thereby strengthening detoxification capacity and restoring redox homeostasis (11). Beyond canonical redox buffering, accumulating evidence—particularly in rheumatoid arthritis (RA)—indicates that Nrf2 can restrain NF-κB–dependent inflammatory programs and influence disease-relevant cellular behaviors, including fibroblast-like synoviocyte activation, macrophage polarization, and regulated cell-death pathways such as apoptosis, via modulation of downstream effectors (e.g., heme oxygenase-1, mitogen-activated protein kinase) and coordination with the RA immune microenvironment (12, 13).However, most existing reviews either focus on a single arthritis subtype or primarily catalogue Nrf2 activators, and therefore rarely provide a cross-disease, cell-type–resolved synthesis that links Nrf2 activity to joint-niche features (e.g., dominant ROS sources, stromal–immune crosstalk, cartilage versus bone involvement, regulated cell-death programs, and immunometabolic stress) across RA, PsA, and AS.

Accordingly, a cross-disease framework is needed to delineate which components of the ROS-inflammation circuit are shared versus subtype-specific, and to distinguish Nrf2-dependent checkpoints from parallel Nrf2-independent mechanisms. Importantly, the joint microenvironment—encompassing synovium, cartilage, subchondral bone, and infiltrating immune compartments—differs substantially among RA, PsA, and AS, and this heterogeneity is likely to determine the context-dependent outcomes of Nrf2 modulation. Moreover, while a range of synthetic and natural-product Nrf2 activators has shown promising preclinical activity, translation remains constrained by limited bioavailability, potential off-target effects, and the lack of mechanism-based biomarkers to monitor pathway engagement *in vivo*.

Here, we systematically review the regulatory roles of Nrf2 across the arthritis autoimmunity-autoinflammation spectrum—RA, PsA, and AS—with an emphasis on how Nrf2 interfaces with core autoimmune mechanisms (antigen presentation, autoreactive cell activation, immune tolerance) within the microenvironmental ROS-inflammation circuit. We summarize common mechanistic themes and subtype-specific differences, map Nrf2-dependent nodes across redox, inflammatory, and immunometabolic pathways, and integrate emerging evidence on pharmacologic and natural Nrf2 activators with advances in joint-targeted delivery platforms. Finally, we discuss key translational challenges and highlight future directions for developing Nrf2-centered, multi-targeted therapeutic strategies for arthritis.

## 2 Overview of the Nrf2 signaling pathway

Nrf2 is a basic leucine zipper (bZIP) transcription factor central to cellular antioxidant and anti-inflammatory defenses (14). The pathway comprises the transcription factor Nrf2, its repressor Kelch-like ECH-associated protein 1 (Keap1), and antioxidant response elements (AREs) in target-gene promoters (15). Under basal conditions, Keap1 promotes ubiquitin–proteasome–dependent turnover of Nrf2, thereby limiting ARE-driven transcription (16). Structurally, Nrf2 contains seven Neh domains: Neh2 (DLG/ETGE motifs) mediates Keap1 binding; Neh1 harbors the bZIP region required for DNA binding; Neh4/5 recruit co-activators such as CBP/p300; and Neh3 supports chromatin remodeling to drive target-gene transcription (17, 18).

Upon oxidative or electrophilic stress, key cysteine residues in Keap1 (e.g., Cys151, Cys273, and Cys288) are modified, weakening Keap1–Cullin3 E3 ligase activity and allowing newly synthesized Nrf2 to accumulate (19). Stabilized Nrf2 translocates to the nucleus, heterodimerizes with small Maf proteins, and binds AREs to induce cytoprotective genes (e.g., HO-1 and NQO1) (17). Genetic and CRISPR–Cas9 studies support this causal pathway: loss of Nrf2 aggravates oxidative injury, whereas Keap1 loss-of-function or Nrf2 gain-of-function enhances antioxidant defenses (20).

Under chronic arthritic stress, excess ROS and inflammatory cues mutually reinforce each other, amplifying oxidative injury and cytokine signaling (21). Inflammatory mediators further boost ROS through NOX activation, mitochondrial dysfunction, and metabolic stress, thereby sustaining a feed-forward inflammatory milieu (22). In arthritic joints, Nrf2 functions as a counter-regulatory “brake”: oxidative/electrophilic stress modifies Keap1 cysteines, stabilizing Nrf2 and enabling ARE-driven transcription of antioxidant and detoxification genes that can limit ROS accumulation and attenuate downstream inflammatory amplification (23, 24). Therefore, precise modulation of Nrf2 activity represents a promising therapeutic strategy for mitigating oxidative damage, controlling inflammation, and maintaining tissue homeostasis (25). Beyond canonical antioxidant defense, Nrf2 is increasingly implicated in immunoregulation: it can shape dendritic-cell maturation and antigen-presentation programs, tune pro-inflammatory cytokine outputs, and thereby influence autoreactive lymphocyte priming and inflammatory amplification (26)—providing a conceptual link between redox control and autoimmune pathology. With the core regulatory logic of the Keap1–Nrf2 axis in mind, we next discuss how Nrf2 outputs manifest in distinct arthritis subtypes, including RA, PsA, and AS, and how these outputs intersect with subtype-specific inflammatory and redox microenvironments.

## 3 Mechanistic roles of Nrf2 in different arthritis subtypes

### 3.1 RA

RA is a systemic autoimmune disease characterized by chronic, symmetrical synovitis, with clinical manifestations including joint swelling, morning stiffness, pain, and restricted mobility (27); in advanced stages, it leads to bone erosions, joint deformities, and extra-articular involvement, particularly of the cardiovascular and pulmonary systems. RA affects approximately 0.46% of the global population, with a higher incidence in women, especially those aged 20–40 years (28). Pathogenesis is initiated by aberrant activation of autoreactive T and B cells and production of autoantibodies such as anti-citrullinated protein antibodies (ACPAs), resulting in immune complex deposition and synovial inflammation. In this autoimmune setting, impaired redox control may further facilitate the breakdown of self-tolerance by promoting ROS-driven neoantigen generation (e.g., enhanced protein modification/citrullination), augmenting antigen-presentation and costimulatory programs, and amplifying autoreactive lymphocyte priming and persistence (25). Activated macrophages and fibroblast-like synoviocytes (FLS) then sustain high levels of TNF-α, IL-1β, and IL-6, driving NF-κB and NLRP3 activation, pannus formation, cartilage degradation, and osteoclast-mediated bone resorption (29, 30).

**Figure 2:**
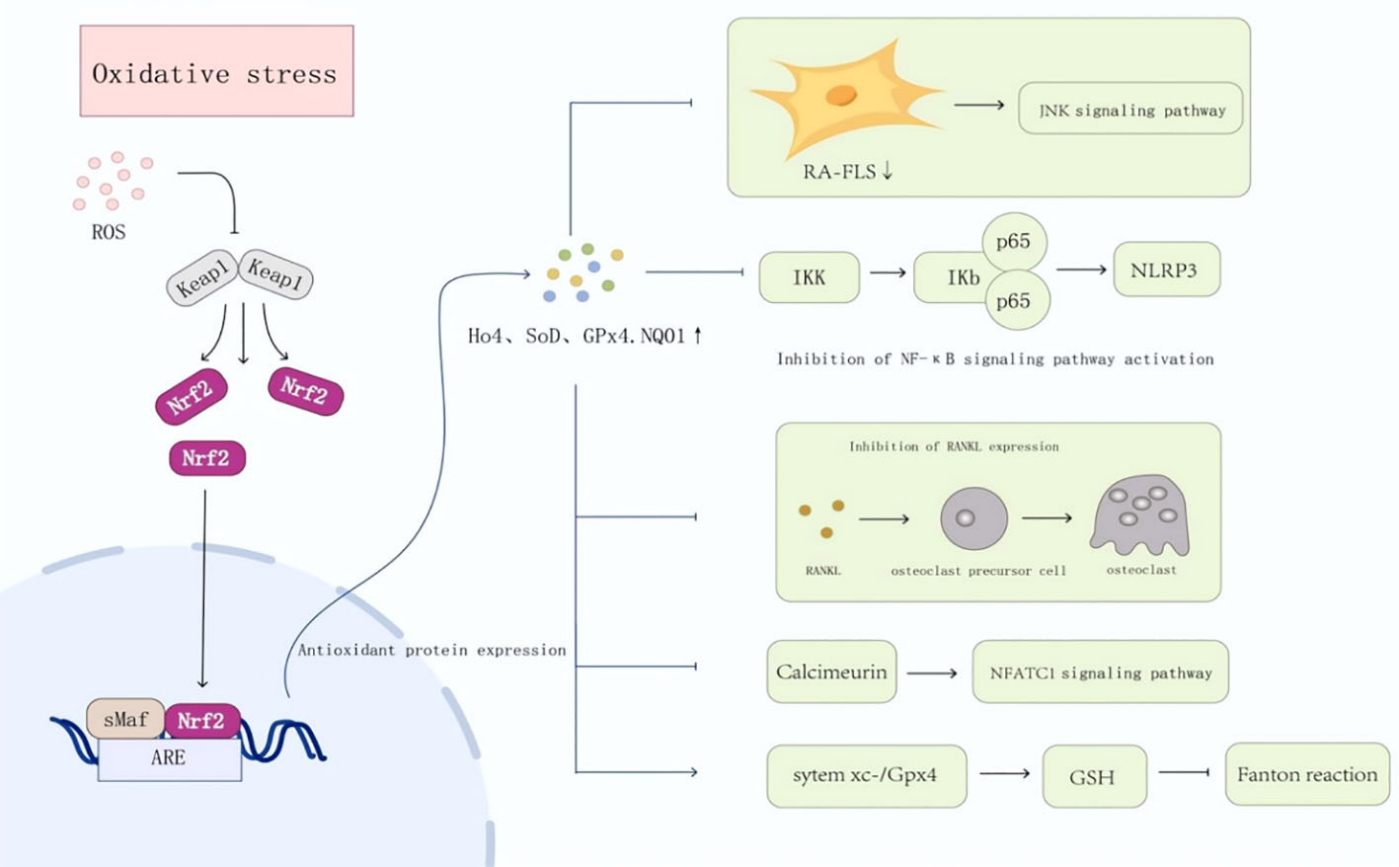
Oxidative stress and Nrf2 signaling pathway in the pathogenesis of rheumatoid arthritis (RA) In RA, excessive ROS activates Nrf2 by inducing Keap1 conformational changes; activated Nrf2 binds ARE to transcribe HO-1, SOD, GPX4, and NQO1, which inhibit JNK/NF-κB/NLRP3 pathways in RA-FLS, suppress osteoclast differentiation via RANKL reduction, and enhance GSH via system xc^−^/Gpx4 to alleviate pathology. RA, rheumatoid arthritis; ROS, reactive oxygen species; Nrf2, nuclear factor erythroid 2-related factor 2; Keap1, Kelch-like ECH-associated protein 1; ARE, antioxidant response element; RA-FLS, rheumatoid arthritis fibroblast-like synoviocytes; NF-κB, nuclear factor-kappa B; Ho-1, heme oxygenase-1; SOD, superoxide dismutase; GPX4, glutathione peroxidase 4; NQO1, NAD(P)H quinone oxidoreductase 1; NLRP3, NOD-like receptor family pyrin domain containing 3; RANKL, receptor activator of nuclear factor-κB ligand; NFATC1, nuclear factor of activated T-cells cytoplasmic 1; GSH, glutathione.

Oxidative stress is a key pathological feature of the RA joint microenvironment and contributes to synovitis and structural damage (24). In RA synovium, excess ROS promotes lipid peroxidation and DNA/protein injury in synoviocytes and chondrocytes, activates redox-sensitive pathways (e.g., NF-κB/MAPK and inflammasomes), and enhances production of cytokines and matrix-degrading enzymes (31). Rather than reiterating the general oxidative stress–inflammation cycle (**Figure 1**), we focus below on how Nrf2 rewires cell-type–specific programs to counter this RA-specific oxidative–inflammatory milieu and to intersect with core autoimmune processes such as inflammatory antigen presentation, autoreactive effector amplification, and failure of resolution (**Figure 2**).

Even so, a key question is why RA persists despite Nrf2 acting as an endogenous brake on ROS-induced inflammation. A key contributing factor is a “ROS–Nrf2 mismatch,” whereby the chronic oxidative/inflammatory burden in the hypoxic synovial pannus exceeds the magnitude and durability of endogenous Nrf2-mediated cytoprotection. Additionally, Nrf2 signaling may be functionally insufficient in a cell- and stage-dependent manner (e.g., dysregulated Keap1–Nrf2 interaction, impaired nuclear translocation, exhausted antioxidant reserves, or metabolic constraints), such that Nrf2 engagement may not fully restore redox homeostasis. Finally, RA is sustained by Nrf2-independent drivers, including immune complex– and cytokine-driven feed-forward loops, persistent antigen presentation, and stromal–immune crosstalk, which can maintain synovitis even with partial Nrf2 activation. These considerations highlight a potential limitation of Nrf2-targeted therapy: in established RA, global Nrf2 activation may not overcome local pathway insufficiency and dominant inflammatory circuits. This supports the need for joint-targeted delivery, biomarker-guided patient selection (functional Nrf2 signatures overexpression alone), and combination strategies to widen the therapeutic window. Below, we detail how Nrf2 intersects with key pro-inflammatory signaling pathways in RA, which may help address these sources of functional insufficiency.

Against this background, Nrf2-mediated negative regulation of key inflammatory cascades—including NF-κB, STING, and the NLRP3 inflammasome—represents a central mechanism by which Nrf2 helps uncouple oxidative stress from chronic inflammation. Viewed as a redox checkpoint, Nrf2 may also shape the autoimmune synovial niche by limiting inflammatory antigen-presentation outputs and restraining self-sustaining immune amplification (32). In the following sections, we summarize how Nrf2 modulates these pathways to disrupt the self-amplifying “ROS–inflammation–tissue destruction” loop in RA.

#### 3.1.1 Nrf2 regulation of pro-inflammatory signaling pathways

In RA, oxidative stress and inflammation are tightly coupled, jointly promoting synoviocyte activation, aberrant immune cell polarization, and osteoclastogenesis. Excessive ROS and reactive nitrogen species (RNS) damage joint-resident cells and are associated with activation of key pro-inflammatory pathways—including NF-κB and the NLRP3 inflammasome—and have been linked to engagement of DNA-sensing programs such as cGAS–STING, sustaining high levels of TNF-α, IL-1β, and IL-6. Persistent cytokine release further amplifies ROS generation, driving a self-perpetuating “ROS–inflammation” loop that underlies chronic disease progression. Importantly, Nrf2-mediated suppression of these pathways is not unique to RA, but rather reflects a conserved anti-inflammatory program established across diverse inflammatory settings. Seminal work has shown that Nrf2 can dampen NF-κB-driven inflammation by limiting oxidative cues that promote IκBα degradation and p65 nuclear translocation and, in some contexts, through reported molecular crosstalk with NF-κB components across multiple inflammatory models (33). Likewise, Nrf2–STING antagonism was initially characterized in antiviral and sterile inflammatory responses, where Nrf2 activation constrains excessive type I IFN–linked inflammation (34). In the RA synovial niche—where FLS and synovial macrophages are central pathogenic effectors—this conserved regulatory logic is particularly relevant, as Nrf2-driven antioxidant and stress-adaptation programs can limit ROS accumulation and thereby restrain redox-sensitive inflammatory outputs in these cell types. Specifically, Nrf2 activation in RA-FLS has been associated with reduced pathogenic proliferation and decreased secretion of matrix-degrading enzymes (e.g., MMPs) and pro-inflammatory cytokines (35), while in synovial macrophages it dampens TNF-α/IL-1β production that fuels inflammatory cell–cell crosstalk, osteoclastogenesis, and sustained synovitis (36). Collectively, these Nrf2-dependent restraints can attenuate synovitis and osteoclast-mediated bone erosion and may weaken self-sustaining immune amplification within the autoimmune synovial microenvironment by reducing cytokine milieus that support pathogenic effector programs.

##### 3.1.1.1 Inhibition of the NF-κB pathway

NF-κB is one of the most prominently activated inflammatory cascades in RA synovium, driving expression of TNF-α, IL-1β, IL-6, and COX-2 and establishing an inflammatory amplification circuit that sustains chronic synovitis. Building on conserved anti-inflammatory mechanisms described across inflammatory contexts (10, 23), Nrf2 inhibits NF-κB activation primarily through redox control: induction of antioxidant enzymes such as HO-1, SOD, CAT, and GPx scavenges ROS/RNS, lowers oxidative stress burden, and interrupts ROS-driven phosphorylation and degradation of IκB-α, thereby limiting nuclear translocation of the NF-κB p65 subunit (37–39). In RA, this restraint is especially relevant in key pathogenic compartments—fibroblast-like synoviocytes (FLS) and synovial macrophages—where NF-κB signaling directly supports synovial hyperplasia, cytokine amplification, and cartilage-destructive programs. In addition, Nrf2 can negatively modulate downstream NF-κB-linked signaling axes (e.g., JAK2/STAT3, MAPK, PI3K/Akt), further suppressing transcription of pro-inflammatory cytokines and tissue-destructive mediators (40–42). Together, reduced oxidative burden and restrained NF-κB signaling contribute to the mitigation of synovial inflammation and cartilage damage in RA, and may indirectly constrain pathogenic immune polarization by weakening cytokine circuits that sustain autoreactive effector responses within inflamed synovium (43).

##### 3.1.1.2 Suppression of the cGAS–STING pathway

The stimulator of interferon genes (STING) pathway senses cytosolic double-stranded DNA (dsDNA) and triggers type I interferon (IFN-I) and pro-inflammatory cytokine release (44). Mechanistic studies in antiviral and sterile inflammatory settings have reported that Nrf2 activation can dampen STING–IFN signaling, positioning Nrf2 as a general brake on excessive DNA-sensing–driven inflammation (45). In RA, synovial tissues show evidence of DNA damage and aberrant STING activation, which has been implicated in sustaining inflammatory remodeling and immune activation and may contribute to T-cell, B-cell, and osteoclast dysfunction (46). In this synovial context, available data support an indirect, redox-dependent model in which Nrf2 limits ROS-driven oxidative DNA damage and reduces the pool of immunostimulatory self-DNA (47), thereby lowering cGAS–STING engagement and downstream IFN-I outputs and weakening STING-linked inflammatory remodeling in RA synovium. Notably, although Nrf2 activation has been reported to coincide with reduced STING pathway activity (and, in some settings, altered STING expression) (34), direct transcriptional regulation of STING by Nrf2 has not been definitively established and may reflect secondary remodeling of cellular stress programs. Consistently, Nrf2 deficiency has been linked to sustained STING activation and aggravated joint pathology in arthritis models, supporting functional relevance of Nrf2–STING antagonism in the RA setting (48). However, direct patient-tissue validation at cell-type resolution remains limited, highlighting the need for human synovial studies integrating Nrf2 perturbation or activity readouts with cGAS–STING pathway outputs.

##### 3.1.1.3 Inhibition of NLRP3 inflammasome activation

The NLRP3 inflammasome, highly expressed in RA synovium, promotes caspase-1–dependent maturation and release of IL-1β and IL-18, contributing to synovitis, pannus formation, and osteoclastogenesis (49). Oxidative stress is a major trigger of NLRP3 activation (50). By enhancing cellular antioxidant capacity and reducing mitochondrial ROS, Nrf2 dampens NLRP3 inflammasome activation (51). Nrf2 also preserves mitochondrial function, limits mitochondrial DNA leakage and ATP release, and thereby indirectly inhibits NLRP3 assembly and activation, ultimately ameliorating synovial inflammation and pain in RA (52, 53). Given that IL-1 family cytokines reinforce myeloid activation and inflammatory effector circuits, Nrf2–NLRP3 antagonism may further weaken self-perpetuating immune amplification in RA synovium.

#### 3.1.2 Nrf2 suppression of osteoclast overactivation

Osteoclasts are multinucleated cells derived from the monocyte–macrophage lineage that resorb mineralized bone matrix via V-ATPase–mediated acid secretion and cathepsin K activity, thereby supporting physiological bone remodeling and mineral homeostasis (54). In RA, osteoclast activity is markedly upregulated and represents a central mechanism of bone loss and joint destruction (55). Pro-inflammatory cytokines such as TNF-α, IL-1β, and IL-17 induce receptor activator of nuclear factor-κB ligand (RANKL) expression and enhance RANK–RANKL signaling, driving sustained osteoclast differentiation and activation. Concurrently, oxidative stress is significantly elevated in RA lesions; ROS function as critical second messengers that activate NF-κB and nuclear factor of activated T cells 1 (NFATc1) and potentiate RANKL-induced signaling, thereby promoting osteoclastogenesis and resorptive capacity and accelerating bone erosion (56–58). Thus, inflammatory cytokines and ROS synergistically drive pathological osteoclast activation in RA.

Nrf2 counteracts this process by integrating antioxidant and anti-inflammatory regulation. Activation of Nrf2 induces downstream antioxidant enzymes—including HO-1, NQO1, SOD, and GPx—enhancing ROS scavenging, maintaining cellular redox homeostasis, and suppressing ROS-driven NFATc1 induction, which collectively attenuates osteoclast differentiation and bone resorption (59). In parallel, Nrf2 limits NF-κB activation and reduces expression of pro-inflammatory cytokines, indirectly weakening RANKL signaling and dampening inflammation-driven osteoclastogenesis (33, 60). Experimental studies further show that activation of the p62/Nrf2 pathway strengthens antioxidant defenses and blocks RANKL-induced ROS generation, effectively inhibiting osteoclast hyperfunction and structural bone damage in RA (61–63). Together, these findings indicate that Nrf2 restrains osteoclast overactivation through coordinated control of ROS and inflammatory pathways and represents a promising molecular target for mitigating RA-associated bone erosion.

#### 3.1.3 Nrf2 counteracts RA-FLS pathological activation

Under physiological conditions, FLS maintain synovial homeostasis by secreting synovial fluid and regulating ECM turnover, thereby supporting joint lubrication and structural integrity (64). In RA, persistent inflammatory stimuli and oxidative stress drive FLS to adopt a pathogenic RA-FLS phenotype characterized by aberrant proliferation, migration, invasiveness, and pro-inflammatory activity. Excessive ROS generation in RA-FLS activates NF-κB and MAPK signaling, augments inflammatory mediator production, and promotes aggressive cellular behavior. Activated RA-FLS secrete pro-inflammatory cytokines (e.g., IL-6, IL-8), matrix-degrading enzymes (e.g., MMP-1, MMP-3), and osteoclast-activating factors (e.g., RANKL), thereby sustaining synovitis, promoting cartilage degradation, and contributing to bone erosion (65). This ROS-rich, cytokine-amplifying milieu makes Nrf2 an endogenous counter-regulatory node; below, we summarize how Nrf2 restrains RA-FLS pathogenic programs while restoring redox balance.

Activation of the Keap1/Nrf2/HO-1 axis upregulates antioxidant enzymes such as SOD, HO-1, and GSH-Px, enhances ROS clearance in the synovial microenvironment, and alleviates oxidative damage (66). This antioxidant response suppresses abnormal RA-FLS proliferation (via Cyclin D1 downregulation), reduces adhesion (through inhibition of integrin α5β1 signaling) and limits epithelial–mesenchymal transition by downregulating Snail and Vimentin, thereby mitigating pathological synovial remodeling (67–70). Beyond redox control, Nrf2 modulates inflammatory signaling in RA-FLS by reducing IKKβ phosphorylation, which lowers TNF-α, IL-6, IL-33, MMP-1, and MMP-3 expression and consequently restrains cartilage degradation and inflammatory propagation (71). In addition, Nrf2 upregulates TRIB1 and suppresses JNK phosphorylation, limiting RA-FLS migration and invasiveness, while attenuation of ERK-dependent proliferative signaling further curbs pathogenic synoviocyte expansion (72).

From a translational perspective, pharmacologic and genetic strategies targeting the Nrf2 axis show promising effects on RA-FLS. The CYP2E1 inhibitor Q11 promotes Nrf2 nuclear translocation and HO-1 expression, concurrently inhibiting ROS production and pro-inflammatory cytokine release and suppressing RA-FLS proliferation and migration (73). Overexpression of the long non-coding RNA LINC00638 similarly activates the Nrf2/HO-1 pathway, reverses pathological RA-FLS activity, and alleviates synovial inflammation in experimental models (74). Collectively, these findings indicate that Nrf2 orchestrates a coordinated antioxidant, anti-inflammatory, and anti-proliferative program that restrains RA-FLS pathogenic activation and may help limit synovial hyperplasia and joint destruction in RA.

#### 3.1.4 Nrf2 in cartilage degeneration and chondrocyte homeostasis

Cartilage degeneration is a major structural hallmark of RA in addition to synovitis and bone erosion (75). Within the inflamed joint, pro-inflammatory cytokines (e.g., TNF-α and IL-1β) and ROS synergize to drive chondrocyte stress responses, increase catabolic enzymes (notably MMPs and aggrecanases such as ADAMTS), and accelerate extracellular matrix (ECM) breakdown, thereby promoting progressive cartilage loss (76).

In this context, the Keap1–Nrf2 axis is highly relevant to cartilage homeostasis in RA. Nrf2 activation in chondrocytes induces antioxidant and cytoprotective programs (including HO-1/NQO1/GCLC-related outputs), restrains redox-sensitive inflammatory pathways (e.g., NF-κB/MAPK), and has been associated with reduced ECM-degradative signaling and chondrocyte injury (77).

Importantly, experimental evidence supports a protective role of Nrf2 against joint and cartilage destruction, as Nrf2 deficiency has been linked to exacerbated cartilage damage, while pharmacological Nrf2 activation (e.g., sulforaphane) has been proposed to confer joint-protective effects (78, 79).

Collectively, these findings suggest that Nrf2 regulation may influence the balance between cartilage degeneration and reparative capacity in RA, complementing its established roles in modulating synovial inflammation and osteoclast-driven bone erosion.

#### 3.1.5 Nrf2-mediated reprogramming of macrophage polarization

Macrophages are core effector cells of the innate immune system and participate in immune surveillance, inflammatory responses, and tissue remodeling (80). In the RA synovium, synovial macrophages are major drivers of chronic inflammation and joint destruction. Pro-inflammatory macrophage activation programs are enriched and continuously release TNF-α, IL-1β, IL-6, and MCP-1, thereby activating T cells, FLS, and osteoclasts and sustaining tissue damage (81). This pro-inflammatory program is accompanied by increased ROS generation, which induces oxidative stress, activates inflammatory pathways such as NF-κB and NLRP3, and is associated with a shift toward often glycolytic, activation-linked immunometabolic reprogramming that can further amplify inflammatory outputs (82, 83). Beyond local tissue injury, such redox-inflamed macrophage states may also reinforce autoimmune amplification by sustaining cytokine milieus that favor pathogenic effector responses within the synovial niche (84, 85).

Importantly, Nrf2-dependent coupling of redox control to metabolic remodeling is not unique to RA. Across diverse inflammatory settings, Nrf2 acts as a stress-adaptation hub that can shape mitochondrial ROS handling and the balance between glycolysis and oxidative metabolism, thereby influencing inflammatory effector functions and cytokine production (16). In RA, this conserved regulatory logic appears particularly relevant in synovial macrophages, where Nrf2 activation engages antioxidant and anti-inflammatory programs that limit ROS-dependent inflammatory signaling and restrain synovitis-associated tissue damage. Specifically, activation of the Nrf2/HO-1 axis upregulates antioxidant enzymes (e.g., SOD and GPx), enhances intracellular ROS scavenging, and thereby limits ROS-fueled NF-κB activation and reduces TNF-α and IL-1β release (86). Nrf2 has also been reported to downregulate NLRP3 inflammasome components and inhibit caspase-1 activation, restricting IL-1β maturation and the propagation of synovial inflammation (87). Consistent with its broader metabolic functions, Nrf2 can attenuate activation-linked glycolytic remodeling in pro-inflammatory macrophages, dampening inflammatory potential (88). By curbing these dominant pro-inflammatory outputs in the RA synovial microenvironment, Nrf2 may indirectly reduce inflammatory “licensing” signals that sustain autoreactive T-cell persistence and stromal pathogenicity (89, 90).

Beyond restraining pro-inflammatory activation, Nrf2 also supports anti-inflammatory, resolution/tissue-repair–associated macrophage programs. Activation of the Nrf2/SIRT3/SOD2 axis reduces mitochondrial ROS and promotes anti-inflammatory mediators such as IL-10, supporting the establishment and maintenance of anti-inflammatory macrophage states (91). The Keap1–Nrf2–IL-10 axis has been linked to increased IL-10 alongside reduced TNF-α and IL-6, thereby alleviating local inflammation and joint damage (92). Moreover, Nrf2/HO-1 activation has been reported to shift macrophage programs toward anti-inflammatory, tissue-repair–associated states while attenuating oxidative stress in the synovial microenvironment—for example, by inhibiting LPS-induced ROS production—thereby helping to restrain bone erosion (93). These anti-inflammatory macrophage programs can further contribute to immune regulation through IL-10 and other pro-resolving mediators that may limit effector T-cell amplification and support regulatory networks (including Treg-associated functions), forming a negative-feedback circuit that helps restrain autoimmune escalation in RA synovium. In addition, Nrf2 has been implicated in preserving GPX4 activity, suppressing lipid peroxidation, and limiting ferroptosis in macrophages, potentially conferring synergistic cytoprotective effects within RA lesions and further reducing tissue injury (94–96).

#### 3.1.6 Nrf2 in immunometabolic reprogramming and trained immunity

Beyond balancing pro- and anti-inflammatory programs, Nrf2 modulates macrophage immunometabolism and may shape the persistence of synovitis in RA (97). Within the hypoxic and cytokine-rich RA joint, macrophages adopt a glycolytic, ROS-rich inflammatory state in which metabolic rewiring and inflammatory signaling become tightly coupled. Nrf2 operates at this redox–metabolic interface by coordinating glucose partitioning, mitochondrial ROS handling, and defenses against lipid peroxidation, thereby potentially influencing both the magnitude and durability of inflammatory macrophage responses (5).

Mechanistically, one convergent route links glucose utilization to antioxidant capacity through a PPP–NADPH–GSH axis (98). In inflammatory macrophages, enhanced glycolysis supports energetic and biosynthetic needs, while diversion of glucose flux through the pentose phosphate pathway can increase NADPH availability and sustain glutathione synthesis/recycling (98). This “PPP→NADPH→GSH” coupling supports GPX4-dependent detoxification of lipid hydroperoxides (99), providing a plausible mechanism by which Nrf2-associated glucose partitioning constrains lipid-peroxidation stress, ferroptosis-linked injury, and downstream inflammatory amplification in the synovium (100). In parallel, pro-inflammatory activation remodels the TCA cycle and elevates mitochondria-derived ROS (mtROS), with inflammatory metabolites such as succinate reinforcing inflammatory outputs (101). Nrf2 has been implicated in strengthening mitochondrial redox buffering and quality control, thereby limiting mtROS and attenuating downstream inflammatory signaling. Conversely, inflammation-derived metabolites—most notably itaconate—can alkylate KEAP1 and activate Nrf2, creating bidirectional coupling between metabolic state and Nrf2-dependent transcriptional regulation (102).

A critical evidence caveat is that, in RA, much of the current support for Nrf2-driven immunometabolic rewiring (e.g., glycolysis–PPP coupling and TCA remodeling) remains associative, and direct causal demonstrations (32)—such as Nrf2 loss-of-function abrogating these metabolic shifts in primary RA synovial macrophages *in vivo*—are still limited. Accordingly, the precise hierarchy and necessity of Nrf2 within chronic inflammatory metabolic networks remain to be defined (13).

These redox–metabolic interactions also suggest potential links to trained immunity, a durable form of innate immune “memory” underpinned by coordinated metabolic shifts (often toward aerobic glycolysis) and epigenetic remodeling (103, 104). Given its roles in regulating NADPH/GSH homeostasis, mtROS, and metabolite sensing (e.g., itaconate–KEAP1 signaling), Nrf2 is positioned to influence the establishment or persistence of trained inflammatory set points in chronic synovitis (105, 106). From a translational perspective, synovial heterogeneity in hypoxia, mitochondrial stress, lipid-peroxidation burden, and metabolite availability is expected to shape how effectively Nrf2 modulation can reprogram inflammatory macrophage programs across RA subsets (107–109), supporting biomarker-guided strategies (e.g., glycolysis/redox/lipid-peroxidation signatures and Nrf2 target outputs) to identify endotypes most likely to benefit.

#### 3.1.7 T cell–intrinsic effects of Nrf2 on autoreactivity and IL-23/IL-17 axis

Accumulating evidence indicates that Nrf2 can influence RA-related adaptive immunity through dual mechanisms: T cell–extrinsic effects (reshaping cytokine milieus generated by APCs/myeloid cells or stromal cells that condition T-cell activation) and T cell–intrinsic regulation (direct modulation of TCR-linked activation and differentiation programs). Genetic and pharmacological studies support that Nrf2 can tune TCR signaling thresholds and immunometabolic fitness, thereby influencing CD4^+^ T-cell expansion and effector lineage commitment (110). While systemic Nrf2 activation has been reported to alleviate severe autoimmune inflammation in preclinical models (26), T cell–specific manipulation studies more directly and unequivocally demonstrate a cell-intrinsic role of Nrf2 in constraining activation-driven T-cell responses.

In arthritis-relevant settings, Nrf2 activation also confers benefit in T cell–mediated autoimmune arthritis models (e.g., SKG mice), where pathogenic Th17 expansion and IL-17 production are key features (111). In selected contexts, Nrf2 has been linked to modulation of the Th17/Treg balance, including dampening RORγt-associated programs and supporting Foxp3-associated regulatory programs via redox-dependent and -independent mechanisms (112). Nevertheless, Nrf2 effects on T-cell programs appear context-dependent, varying with activation state, metabolic constraints, and local inflammatory/redox cues. Collectively, these data suggest that Nrf2 may modulate pathogenic T-cell responses in RA, through both cell-intrinsic and microenvironment-mediated mechanisms.

Collectively, by coordinately regulating macrophage antioxidant defenses and the balance between pro-inflammatory and anti-inflammatory programs, Nrf2 mitigates inflammation-mediated tissue damage in RA and represents an attractive target for therapeutic modulation of the synovial immune microenvironment.

### 3.2 PsA

**Figure 3:**
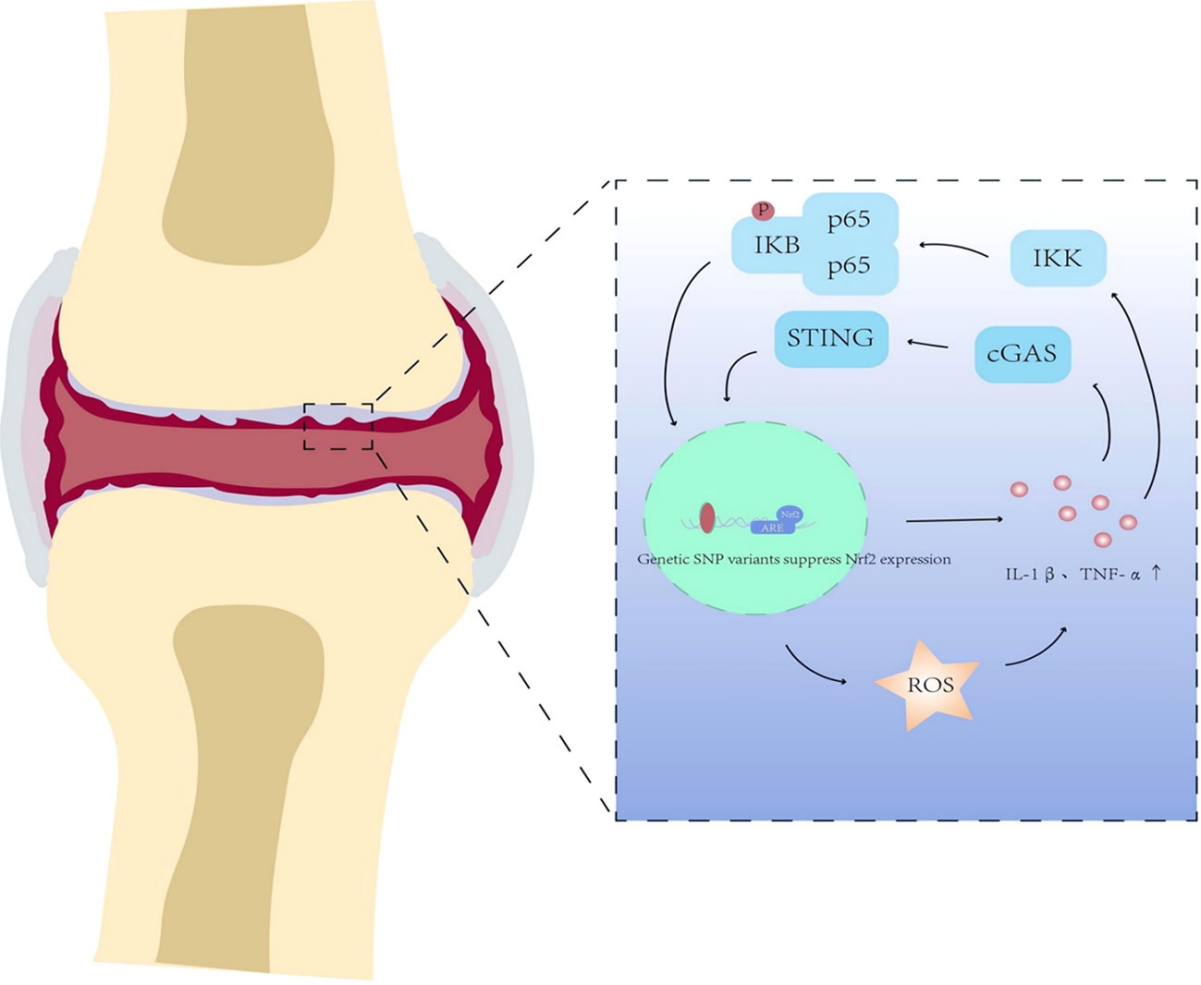
Oxidative stress-mediated activation of the cGAS-STING inflammatory signaling pathway in psoriatic arthritis (PsA). In PsA, ROS promotes activation of the cGAS–STING pathway, leading to IKK-dependent NF-κB activation (p65 phosphorylation and nuclear translocation) and increased production of pro-inflammatory cytokines, including IL-1β and TNF-α. These inflammatory outputs can further enhance oxidative stress and exacerbate joint inflammation and tissue destruction. PsA, psoriatic arthritis; ROS, reactive oxygen species; cGAS, cyclic GMP-AMP synthase; STING, stimulator of interferon genes; NF-κB, nuclear factor-kappa B; IKK, IκB kinase; IL-1β, interleukin-1 beta; TNF-α, tumor necrosis factor-alpha.

Psoriatic arthritis (PsA) is a chronic systemic immune-mediated arthropathy that lies along an autoinflammation–autoimmunity continuum, closely associated with psoriatic skin lesions and classified within the spectrum of seronegative spondyloarthropathies (113). It shows marked clinical heterogeneity, affecting peripheral joints, the axial skeleton, and entheses, and is characterized by joint pain, swelling, dactylitis, and enthesitis (114). In most patients, arthritis develops 5–10 years after the onset of cutaneous psoriasis, although joint disease may occasionally precede skin involvement. Mechanistically, psoriatic skin inflammation can act as a priming site for systemic immune activation: keratinocyte- and neutrophil-associated danger signals and autoantigenic complexes (e.g., LL37-nucleic acid complexes) may drive pathogenic T-cell responses that disseminate to musculoskeletal tissues, supporting a “skin–joint” immune crosstalk axis (115–117). Beyond immune dysregulation, PsA pathogenesis is increasingly recognized to involve pronounced oxidative stress (118), with persistent accumulation of ROS/RNS driving lipid peroxidation, DNA damage, and activation of pro-inflammatory pathways in synovial and osseous tissues. Given this ROS-linked inflammatory circuitry (**Figure 3**), we next discuss whether impaired antioxidant signaling—particularly via Nrf2—contributes to persistence of inflammation and joint damage in PsA. This ROS-linked inflammatory circuitry is tightly coupled to Nrf2-dependent redox regulation, which may act as a brake on immune amplification across skin and joint compartments. In psoriatic skin, impaired Nrf2 signaling may intensify oxidative stress and amplify IL-23/IL-17–skewed cytokine programs, thereby reinforcing systemic immune priming and inflammatory amplification that may propagate to joints (119). Accordingly, restoring Nrf2 activity may enhance antioxidant capacity, dampen redox-driven cytokine escalation, and potentially weaken feed-forward communication between skin and joint inflammation.

Nrf2 plays a pivotal regulatory role in this context. Although Nrf2 protein levels may be relatively elevated in PsA, its functional activity is impaired, as evidenced by reduced expression of downstream antioxidant enzymes (e.g., HO-1) and increased Keap1, indicating functional deficiency of the Nrf2 axis (120). This defect likely contributes to sustained oxidative stress, chronic inflammation, and progressive tissue injury. Oxidative stress is a central pathological driver in PsA (121). Persistent ROS accumulation can exceed endogenous antioxidant capacity and damage lipids, proteins, and DNA. Under physiological conditions, Nrf2 maintains intracellular redox equilibrium by inducing antioxidant enzymes such as GSH-Px, SOD, TrxR, and HO-1, thereby counteracting ROS-induced lipid peroxidation and DNA injury. In PsA, antioxidant capacity is diminished, while lipid peroxidation products such as malondialdehyde (MDA) and 4-hydroxynonenal (4-HNE) are elevated (122). These changes promote covalent protein modification, impaired protein function, cell necrosis/apoptosis, and barrier disruption, facilitating inflammatory mediator infiltration (123). Impaired Nrf2 thus represents a key molecular basis for persistent oxidative imbalance and chronic inflammation, which may create a permissive microenvironment for pathogenic T-cell activation by amplifying danger signaling, inflammatory antigen-handling states, and IL-23/IL-17–skewed cytokine programs (10), rather than directly inhibiting T-cell receptor signaling. In parallel, heightened cutaneous oxidative stress may reinforce IL-23/IL-17–skewed inflammatory circuits and systemic immune priming, providing a plausible redox-sensitive substrate for “skin–joint” immune crosstalk in PsA (119, 124).

Consistent with this view, although PsA-specific evidence for T cell–intrinsic Nrf2 regulation remains limited, emerging genetic and pharmacological studies in broader autoimmune settings suggest that Nrf2 can modulate activation-linked T-cell signaling and immunometabolic fitness, thereby shaping proliferative capacity and effector differentiation. In PsA, these cell-intrinsic mechanisms may complement the more established microenvironment-mediated route described above: oxidative stress–driven activation of APCs/myeloid cells and stromal cells can reinforce IL-23/IL-17–skewed cues that sustain Th17-type effector programs (120, 125, 126). Accordingly, current evidence supports the view that Nrf2 insufficiency in PsA primarily fosters a redox-permissive inflammatory niche that supports pathogenic T-cell amplification. The direct effects of Nrf2 on T-cell activation thresholds, while plausible based on broader autoimmune studies, remain to be conclusively established in PsA-focused studies and are likely context-dependent.

Notably, in PsA, the reported links between Nrf2 dysfunction and immunometabolic perturbations (e.g., glycolytic skewing in myeloid cells) remain largely associative (127). Direct, disease-context validation that Nrf2 activation reverses PsA-relevant metabolic reprogramming—beyond indirectly reshaping redox-sensitive inflammatory outputs—remains limited, underscoring the need for targeted studies integrating Nrf2 perturbation with metabolic flux and single-cell functional readouts.

In parallel, dysfunction of the Nrf2 pathway is also closely associated with aberrant pro-inflammatory cytokine expression in PsA (128). Beyond antioxidant defense, Nrf2 modulates multiple inflammatory cascades. It suppresses NF-κB activation by preventing IκB-α degradation and p65 nuclear translocation, thereby downregulating TNF-α, IL-6, and IL-1β and alleviating synovitis and excessive immune cell activation (129). In addition, Nrf2 negatively regulates the STING pathway, reducing type I interferon and IL-1β production (130). Importantly, STING expression in PsA patients is significantly lower than in RA (48). This lower expression aligns with the view that PsA pathogenesis may rely comparatively less on dominant type I IFN–linked DNA-sensing programs and more on canonical inflammatory axes (e.g., NF-κB) and the IL-23/IL-17 pathway (131). Nevertheless, under conditions of oxidative stress and defective Nrf2 restraint, cGAS–STING sensing of self-DNA released from ROS-injured cells may still contribute to type I IFN–associated inflammatory outputs and synovial/entheseal damage (132). In most PsA cases, however, this mechanism is best viewed as an amplifier rather than a primary disease driver.

Collectively, impaired Nrf2 signaling in PsA may permit oxidative stress to amplify NF-κB–linked cytokine programs and redox-sensitive innate pathways, thereby exacerbating synovitis and structural damage. Viewed through an immune-mediated lens, Nrf2 insufficiency may also strengthen feed-forward communication between inflamed skin and musculoskeletal tissues by sustaining oxidative danger cues and cytokine escalation. A key gap is to define when and where Nrf2 is functionally deficient in patients (despite variable expression levels) and to clarify its interplay with cGAS–STING–related signaling in PsA, which will be essential for biomarker-guided, joint-targeted translation.

### 3.3 AS

Ankylosing spondylitis (AS) is a chronic immune-mediated disorder within the spondyloarthropathy spectrum that primarily affects the sacroiliac joints and spine (133). Clinically, it presents with inflammatory low back pain and morning stiffness that improves with activity, and it may involve peripheral arthritis, enthesitis, and uveitis (134). Although the precise etiology remains unclear, AS is closely associated with genetic susceptibility—particularly HLA-B27—and immune dysregulation (135, 136). Persistent cytokine activation and oxidative stress synergistically drive cartilage destruction and syndesmophyte formation (137). At inflamed sites, infiltrating T cells and macrophages release TNF-α, IL-17, and IL-23, perpetuating inflammation and abnormal bone remodelling (138). Patients also exhibit elevated ROS and impaired antioxidant systems, including reduced SOD and GPx activity (139). Consistent with its position along the autoinflammation–autoimmunity continuum, HLA-B27–linked immune dysregulation and IL-23/IL-17–skewed effector programs are thought to couple tissue-site inflammation to pathological bone remodeling in AS (140, 141).

In AS, oxidative stress not only directly injures entheseal and spinal tissues but also enhances inflammatory signaling via NF-κB and related pathways. This ROS-driven amplification of cytokine production can sustain chronic inflammation and contribute to aberrant bone remodelling (137, 142). Nrf2 dysfunction appears to be a key molecular basis of this imbalance. Mesenchymal stem cells from AS patients show decreased Nrf2 and HO-1 expression, impaired ROS clearance, and increased susceptibility to oxidative injury, promoting sustained ROS accumulation and additional ROS generation from NADPH oxidase and mitochondrial dysfunction (143). Consistently, AS patient plasma displays higher total oxidant status (TOS) and oxidative stress index (OSI), and lower total antioxidant status (TAS) than healthy controls, further implicating elevated oxidative stress and weakened antioxidant capacity in disease pathogenesis (144). This redox–inflammatory imbalance may occur in the context of HLA-B27–associated cellular stress. Mechanistically, HLA-B27 misfolding has been linked to endoplasmic reticulum stress/unfolded protein responses and increased ROS generation, which may reinforce redox-sensitive inflammatory signaling and IL-23/IL-17–biased immune activation. However, while oxidative stress is a plausible upstream cue for Nrf2 engagement, direct evidence establishing a contiguous HLA-B27 misfolding → ROS → Nrf2 causal axis in disease-relevant entheseal/axial compartments remains limited. Accordingly, Nrf2 modulation in AS is best viewed as a working model in which redox stress may contribute to context-dependent Nrf2 insufficiency or maladaptive activation, with downstream implications for inflammatory amplification and bone remodeling that warrant systematic validation. Although Nrf2 has been linked to osteogenic programs (e.g., Runx2/OSX) and major osteoinductive pathways (e.g., BMP/Smad) in other contexts (145), direct mechanistic evidence connecting Nrf2 to these axes in AS-relevant axial/entheseal stromal compartments remains limited, and is therefore best framed as a plausible downstream hypothesis rather than a settled pathway.

Beyond its antioxidant role, Nrf2 modulates inflammatory responses in AS. Local immune activation in axial joints drives the production of TNF-α, IL-17, and IL-23, fueling chronic inflammation and tissue remodeling (135). Experimental evidence indicates that targeted Nrf2 activation can inhibit the ROS/NF-κB axis and ameliorate disease in preclinical models, supporting its therapeutic potential (143). Thus, Nrf2 acts as a key redox-sensitive regulator that can restrain inflammatory signaling in AS.

The reported dual role of Nrf2—anti-inflammatory yet potentially pro-osteogenic—can be reconciled by a compartment-specific model. In immune cells, Nrf2 primarily suppresses ROS–NF-κB–driven cytokine production (146). In contrast, within stromal or osteogenic-lineage cells under certain conditions, Nrf2 activity may be biased toward osteogenic differentiation/signaling, thereby potentially influencing syndesmophyte formation. Given the centrality of the IL-23/IL-17 axis in AS, defining how Nrf2 intersects with these cytokine programs across different cell types and disease stages will be crucial for mechanistic and translational clarity (143, 147, 148).

Importantly, AS-specific causal evidence linking Nrf2 to osteogenic-lineage programming is still limited; therefore, this compartment-specific model should be viewed as a working hypothesis that requires validation using cell-type–specific Nrf2 perturbation and human entheseal/osteogenic tissue analyses (149).

From an immunometabolic perspective, the proposed links between Nrf2 and immunometabolic remodeling in AS remain preliminary, with much inference drawn from related diseases. While metabolic features such as mitochondrial ROS have been discussed in the context of redox imbalance and variable Nrf2 activity, direct causal validation remains limited. Cell-type–specific perturbation studies are needed to determine whether Nrf2 directly regulates these metabolic programs or instead acts downstream of chronic oxidative stress.

The major unresolved issue is the context-dependent effect of Nrf2 on bone remodeling. A central question is how Nrf2 activity in different compartments—entheseal stromal/osteogenic lineages versus immune cells—differentially shapes syndesmophyte formation. Addressing this is essential for translating Nrf2-centered therapies without inadvertently promoting pathological new bone. Future work must test these compartment-specific functions. Translationally, this underscores the need to design spatially or cellularly constrained Nrf2-activation strategies (e.g., through site-biased or inflammation-responsive delivery). The goal is to prioritize anti-inflammatory benefits in immune cells while minimizing potential pro-osteogenic exposure in stromal lineages.

## 4 Cross-disease microenvironment comparison

Inflammatory arthritis subtypes (RA, PsA, and AS) are shaped by distinct microenvironmental triggers, dominant ROS sources, and niche-enriched cellular ecosystems, rendering Nrf2 regulation inherently context dependent. We integrate these subtype-resolved differences into a “ROS source–niche–checkpoint” framework (**Table 1**) to move beyond parallel pathway descriptions and to pinpoint disease-stratified vulnerabilities and translational priorities. Briefly, RA is dominated by a hypoxic synovial pannus in which FLS–macrophage networks sustain ROS through NOX activity and mitochondrial stress, with evidence consistent with insufficient Nrf2 pathway output relative to oxidative load (“ROS–Nrf2 mismatch”). PsA reflects a skin–joint inflammatory axis in which redox and inflammatory cues may arise across compartments, implying that Nrf2 activity should be assessed functionally in both skin and joint rather than inferred from expression alone. AS centers on the enthesis/axial niche where stromal/osteogenic compartments link inflammation to ossification, raising a compartment checkpoint in which anti-inflammatory benefits of Nrf2 modulation may intersect with bone remodeling programs.

**Table 1:** Comparative microenvironment framework across RA, PsA, and AS: dominant ROS sources, niche-resolved Nrf2 activity states, subtype-enriched stromal nodes, key gaps, and actionable implications.

Subtype	RA	PsA	AS
Trigger(s) & niche	Autoimmune synovitis; hypoxic pannus	Skin–joint inflammatory axis; IL-17/IL-23 milieu	Enthesis-driven inflammation; mechanical stress; axial niche
Major ROS sources (where)	NOX (FLS/macrophage); mitochondrial ROS (hypoxia); iron-linked lipid peroxidation	ROS from activated myeloid cells; mitochondrial stress; DNA/innate sensing–linked ROS	Mitochondrial ROS in stromal cells; NOX in immune cells; ROS-osteogenic coupling
Nrf2 state (evidence)	Often functionally insufficient vs oxidative load; cell-type– and stage-dependent heterogeneity (expression may not reflect activity).	Expression may be ↑, but activity is blunted (Keap1↑/HO-1↓ pattern)	Reduced antioxidant capacity reported; Nrf2/HO-1 down in patient MSCs
Enriched populations	Invasive FLS; pro-inflammatory–skewed macrophages; osteoclasts	Synovial myeloid cells + skin-derived cues; keratinocyte-immune crosstalk	Enthesis-resident stromal/osteogenic progenitors; IL-17/IL-23 axis
Distinct checkpoint(s)	Nrf2–HO-1 restrains FLS aggressiveness and osteoclastogenesis; macrophage immunometabolism	ROS–cGAS–STING–NF-κB coupling as a subtype-salient node	Nrf2 may diverge: anti-inflammatory yet potentially pro-osteogenic in stromal lineages
Key gap	Which RA endotypes are “Nrf2-low” vs “compensatory Nrf2-high”?	When is STING functionally relevant in joint vs skin compartments?	How to separate immune vs osteogenic effects (syndesmophytes)?
Actionable insight	Synovium-targeted delivery; combine with cytokine/JAK blockade; monitor synovial Nrf2 signature + lipid peroxidation markers	Dual-site (skin+joint) targeting; PD readouts beyond CRP (local redox + innate-sensing markers)	Compartment-specific modulation (immune-targeted vs stromal-sparing); include bone-formation endpoints and imaging.
Autoimmunity feature gradient	High (prototypic autoimmunity)	Intermediate (autoinflammation–autoimmunity continuum)	Lower (HLA-B27–linked immune dysregulation)
Key immune drivers (examples)	Autoreactive T/B-cell responses; ACPAs; immune complexes; synovial antigen presentation	Skin–joint immune crosstalk; IL-23/IL-17–skewed T-cell programs; cutaneous danger signals/autoantigenic complexes (e.g., LL37-containing complexes)	HLA-B27–associated stress/immune dysregulation; IL-23/IL-17 axis; entheseal/axial niche–driven stromal–immune crosstalk

Nrf2, nuclear factor erythroid 2–related factor 2; ROS, reactive oxygen species; HO-1, heme oxygenase-1; NQO1, NAD(P)H:quinone oxidoreductase 1; SOD, superoxide dismutase; GPX4, glutathione peroxidase 4; NF-κB, nuclear factor-κB; NLRP3, NOD-, LRR- and pyrin domain-containing protein 3.

This comparative matrix yields actionable implications (**Table 1**): (i) endotype-guided selection of “ROS-high/Nrf2-insufficient” niches, (ii) compartment-targeted delivery aligned with dominant effector populations (synovium in RA, skin–joint in PsA, enthesis/axial sites in AS, and (iii) niche-resolved target-engagement biomarkers (Nrf2 target-gene signatures plus oxidative-injury indices) to support dose optimization and combination strategies. Future work should prioritize patient-tissue validation of compartment-specific Nrf2 activity states and develop cell-type-selective modulators capable of maximizing anti-inflammatory benefit while minimizing context-dependent liabilities.

## 5 Translational challenges of Nrf2-mediated regulation of the “oxidative stress–inflammation” axis in arthritis

### 5.1 Nrf2-mediated regulation of the “oxidative stress-inflammation” axis in arthritis: current research analysis

Current research advances, key findings, and existing limitations regarding Nrf2-mediated regulation of the oxidative stress–inflammation axis in arthritis are summarized in **Table 2**.

**Table 2:** Nrf2-mediated regulation of the “oxidative stress-inflammation” axis in arthritis: current research analysis.

Analysis dimension	Main findings	Limitations	Reference
Molecular Mechanism	① Nrf2 activates a battery of antioxidant enzymes such as HO-1, SOD, and GPX4, thereby eliminating excessive ROS and disrupting oxidative stress–mediated joint tissue damage at its origin.② By negatively regulating key pro-inflammatory pathways (e.g., NF-κB, NLRP3 inflammasome), Nrf2 markedly suppresses the production of TNF-α and IL-1β, attenuating local joint inflammation.③ Nrf2 maintains mitochondrial integrity and upregulates GPX4 expression, effectively inhibiting mitochondrial apoptosis and ferroptosis in chondrocytes and delaying cartilage degeneration.④ Nrf2 can shift macrophage activation away from pro-inflammatory programs toward anti-inflammatory, resolution/tissue-repair–associated states, enhance IL-10 production, and thereby remodel the synovial immune microenvironment to restrain inflammatory amplification.⑤ Nrf2 suppresses the pathological activation of RA fibroblast-like synoviocytes and osteoclasts, thereby preventing synovial hyperplasia and bone erosion while preserving joint structural integrity.	Terminal effect: ROS and Nrf2 can cross-regulate to form feedback loops, but current studies show significant heterogeneity in effects and mechanisms; most evidence is derived from animal/cell models, with limited validation in human tissues/systems.	(150, 151)
Subtype-Specific Regulatory Effects	RA: Nrf2 activation has been reported to restrain pathological synovial stromal programs (e.g., inflammatory activation and proliferation of FLS), whereas its direct regulation of adaptive immune programs (B/T-cell–related outputs) appears context-dependent and not fully resolved. PsA/AS: regulatory effects exhibit heterogeneity; mechanistic links between Nrf2 and redox–inflammation crosstalk are emerging, but direct disease-specific causal evidence remains limited.	Across PsA/AS, cellular/nuclear-level mechanisms remain incompletely defined; direct evidence supporting Nrf2 as an upstream regulatory node is limited. Many studies focus on selected immune or stromal cell types (e.g., T cells, monocytes/macrophages, fibroblasts) with insufficient systematic verification at the tissue/organism level and limited patient-tissue validation.	(152–154)
Clinical Biomarker/Phenotype Evidence	Circulating NRF2 protein measured in serum/plasma (most commonly assessed by immunoassays, e.g., ELISA) has been associated with RA disease activity, including correlation with DAS28 scores (reported P values as in the original studies) and with prognosis/inflammatory burden in some cohorts. Importantly, because NRF2 is primarily an intracellular transcription factor, circulating NRF2 protein should be interpreted as a circulating Nrf2-related readout rather than a direct marker of pathway activation. Therefore, where feasible, serum NRF2 measurements should be complemented by activity-based indicators, such as Nrf2 target-gene/target-protein outputs (e.g., HO-1/NQO1/GCLC) in PBMCs, synovial fluid, or synovial tissue, to reflect pathway engagement better.	Most clinical studies are limited by modest cohort sizes and heterogeneity in patient background and treatment. In addition, circulating NRF2 protein abundance is not specific for NRF2 pathway activation, as it may reflect variable cell sources (stress/injury-associated release and/or extracellular vesicle cargo) and is sensitive to pre-analytical variables. Consequently, large multicenter cohorts and activity-based biomarker panels (target-gene signatures and/or functional antioxidant capacity readouts) are needed to validate the clinical utility of Nrf2-related biomarkers in serum/plasma and joint-derived samples.	(155–158)
Therapeutic Effect Evidence	Multiple natural compounds have been reported to modulate Nrf2 signaling in RA (e.g., glabridin, Mesua assamica, perillyl alcohol) and AS (e.g., Cassia twigs, celastrol), showing anti-inflammatory and antioxidant effects in preclinical models. Some studies suggest that combining Nrf2 activators with conventional agents may enhance efficacy, but direct comparative studies dissecting pathway-specific mechanisms remain scarce. Importantly, clinically approved Nrf2-modulating agents (e.g., dimethyl fumarate, DMF) provide translational anchoring demonstrating that systemic Nrf2 engagement is feasible in humans, although arthritis-specific efficacy and biomarker-guided positioning remain to be established.	Most evidence is derived from preclinical studies; few compounds have entered clinical trials. Translation to clinical application requires systematic evaluation of pharmacodynamics, pharmacokinetics, and safety.	(37, 38, 92, 159–166)
Prospective Research Directions	① Clarify whether Nrf2 participates in regulating the fate of synovial/immune cells, especially ferroptosis. The status and regulatory mechanisms of Nrf2 in new bone formation and adipogenesis in AS remain unclear.② Investigate whether Nrf2 can regulate the interaction between different immune cell subsets (e.g., Treg, Th17) and cell death pathways; the “oxidative stress–inflammation–immunity” axis requires further exploration and experimental validation.	Multidimensional, systematic research is needed; more attention should be paid to precision medicine and translational research.	

### 5.2 Safety and mechanistic controversies of Nrf2 activation

Nrf2 activation for arthritis faces critical safety and mechanistic controversies that hinder clinical application. First, it exhibits a “double-edged sword” effect: while suppressing joint inflammation, sustained systemic activation may induce immunosuppression and increase oncogenic risk (167). The optimal therapeutic window, therefore, remains undefined, supporting joint-targeted or localized delivery strategies (**Table 3**). Moreover, the persistence of established RA despite endogenous Nrf2 engagement suggests a “ROS–Nrf2 mismatch,” implying that systemic Nrf2 activation alone may not overcome dominant synovial inflammatory circuits. Accordingly, risk-mitigation principles include: (i) joint-targeted/inflammation-responsive delivery to limit systemic exposure; (ii) lowest-effective and/or intermittent dosing where feasible; (iii) activity-based biomarkers (target-gene/functional redox readouts) for stratification and dosing; and (iv) combination regimens (e.g., with cytokine/JAK blockade) to reduce required Nrf2-agonist exposure.

**Table 3:** Key limitations of Nrf2-targeted interventions in arthritis and corresponding potential solutions.

Major limitation of Nrf2-targeted therapy in arthritis	Potential solution/strategy
Poor bioavailability and short half-life of many Nrf2 activators	Stabilize/optimize scaffolds (e.g., prodrugs; soft electrophiles) to improve exposure. Formulate for persistence (cyclodextrin complexes; nanoformulations; sustained-release depots). Integrate PK–PD: quantify drug/metabolites + Nrf2 engagement readouts. Clinical example: SFX-01 (sulforaphane–α-cyclodextrin; phase II) illustrates a translatable package (168).
Limited joint/cell targeting; diluted joint exposure and off-target activation	Prefer joint-first delivery (intra-articular hydrogels/microparticles) to concentrate local activity. Use synovium- or cartilage-homing or cell-targeted carriers (such as macrophages/, or FLS) to reduce off-target exposure. Apply microenvironment-responsive release (ROS-, enzyme-, or pH-triggered systems).
Safety concerns with chronic systemic Nrf2 activation; unclear therapeutic window	Build composite biomarkers: oxidative injury + antioxidant capacity + Nrf2 target-gene signature (169).
Lack of mechanism-based PK–PD biomarkers to confirm target engagement *in vivo*	Pair mechanism readouts with exposure (drug/metabolites), given inter-individual bioavailability variability (170).
Subtype/stage heterogeneity and context dependence of Nrf2 function	Build composite biomarkers: oxidative injury + antioxidant capacity + Nrf2 target-gene signature. Pair mechanism readouts with exposure (drug/metabolites), given inter-individual bioavailability variability. Develop joint-focused imaging/readouts (e.g., probes/reporters) to track *in vivo* pathway modulation.
Unclear clinical positioning and combination strategies with standard-of-care therapies	Design mechanism-guided combinations with csDMARDs/bDMARDs/JAK inhibitors to widen therapeutic windows. Use biomarker-defined endpoints in early-phase trials to enable dose de-escalation and synergy testing. Prioritize regimens that target both inflammatory drivers and oxidative injury components.

Translational anchoring and clinical safety lessons from DMF. Clinically used Nrf2-modulating agents provide an important translational reference point. Dimethyl fumarate (DMF), approved for moderate-to-severe plaque psoriasis (and relapsing MS), is widely considered to engage the Keap1–Nrf2 axis via electrophilic modification of Keap1 cysteines, demonstrating that systemic Nrf2 engagement is feasible in humans (171, 172). Clinical experience also underscores constraints relevant to arthritis translation: DMF commonly causes gastrointestinal intolerance and flushing, and can be accompanied by lymphopenia, indicating potential infection-related risk and the need for monitoring. Therefore, DMF serves as a feasibility anchor rather than efficacy proof for RA/PsA/AS, and arthritis-directed strategies should prioritize targeted delivery and biomarker-guided dosing. Consistently, direct patient-tissue validation of Nrf2 activity (e.g., synovial target-gene outputs and cell-type–resolved readouts) remains limited (173). In parallel, iron-targeted interventions (e.g., chelation or iron-sequestration approaches) represent a plausible adjunct strategy to limit labile-iron–driven lipid peroxidation; however, translation to arthritis will likely require biomarker-guided patient selection and careful safety monitoring, as systemic iron depletion may increase risks of anemia and infection susceptibility.

Second, Nrf2 may exert subtype-specific effects on bone remodeling: it inhibits osteoclast overactivation in RA but may promote osteogenic differentiation in AS (24, 143), likely reflecting distinct ROS microenvironments, although quantitative thresholds remain unclear. Third, evidence linking Nrf2 to immunometabolic polarization is largely correlative, with limited causal validation, particularly at single-cell resolution. Finally, systemic Nrf2 agonists can require substantial systemic exposure in preclinical settings, increasing off-target toxicity risk, including hepatotoxicity (174). The absence of real-time biosensors for Nrf2 activity further complicates dose optimization, creating a trade-off between local anti-inflammatory efficacy and systemic overactivation.

### 5.3 In-depth analysis of clinical translation bottlenecks

Despite substantial preclinical progress, clinical translation of Nrf2-targeted strategies remains constrained by three long-standing and interlinked bottlenecks: (i) unfavorable pharmacokinetics and dose-limiting toxicity, (ii) insufficient joint targeting with off-target activation, and (iii) limited objective readouts to quantify pathway engagement and clinical response.

#### 5.3.1 Pharmacokinetic limitations: the dilemma of bioavailability and toxicity

Most small-molecule Nrf2 activators display poor oral bioavailability (often <10% for agents such as sulforaphane and andrographolides) owing to extensive first-pass metabolism and rapid systemic clearance, resulting in low effective exposure at joint sites (175). To reach pharmacologically relevant intra-articular concentrations, dose escalation is frequently required, which in turn increases the risk of hepatic injury and systemic immunosuppression (175, 176). This “low exposure–high toxicity” paradox represents a central barrier to clinical translation. In principle, prodrug optimization, long-acting depot formulations, and intra-articular or microenvironment-responsive delivery platforms offer routes to enhance local bioavailability while reducing systemic burden; representative strategies and candidates are detailed in **Table 3**.

#### 5.3.2 Insufficient tissue targeting: off-target activation and safety risks

Current Nrf2-targeted approaches rely mainly on systemic administration, which leads to suboptimal drug accumulation in inflamed joints and disproportionate exposure of non-target organs (175, 177). Joint delivery efficiency often falls short of what is needed for site-selective therapy, while tissues such as the liver and kidneys may experience excessive Nrf2 activation, disturbing local redox homeostasis and raising safety concerns (178). Nanocarrier-based systems and ligand- or peptide-mediated targeting have begun to improve joint selectivity in preclinical models, enhancing synovial and cartilage uptake and prolonging intra-articular retention (179, 180). Conceptually, these joint-focused platforms provide a framework for reconciling efficacy with safety; specific carrier designs, targeting ligands, and proof-of-concept studies are summarized in **Table 3**.

#### 5.3.3 Limitations of efficacy evaluation: subjective assessment versus objective quantification

Efficacy evaluation frameworks for Nrf2-targeted therapy remain poorly adapted to pathway-specific modulation. Symptom-based scales such as the visual analogue scale (VAS) for pain are inherently subjective, with reported variability exceeding 15% (181), and thus introduce uncertainty into estimates of true treatment benefit. Conventional imaging modalities (X-ray, MRI) capture structural damage but are insensitive to early or dynamic changes in oxidative stress and Nrf2 activity and typically lag behind joint pathology. Systemic inflammatory markers (CRP, ESR) are neither sensitive nor specific for local synovitis: pronounced synovial inflammation can occur with normal CRP (182), and Nrf2 activators such as sulforaphane may improve synovial pathology with only modest effects on systemic indices (183). These observations highlight a partial decoupling between local Nrf2 signaling and systemic inflammation and underscore the lack of mechanism-based monitoring tools.

Emerging gene-engineering and chemical-probe technologies suggest potential solutions. Nrf2-reporter constructs and fluorescent probes based on protein–protein interaction or ubiquitination can dynamically report Nrf2 stability and activity in cells and tissues (184), and could in principle be adapted to joint imaging to enable non-invasive assessment of Nrf2 modulation in synovium and cartilage. In parallel, constructing a multidimensional efficacy framework that integrates Nrf2-related imaging readouts with clinical scores and oxidative-stress biomarkers (e.g., 8-OHdG) may allow more quantitative and individualized evaluation of Nrf2-targeted interventions. Representative probe designs, imaging modalities, and composite evaluation schemes are summarized in **Table 3**.

Taken together, the central translational challenge is to achieve sufficient, durable Nrf2 activation within diseased joints while minimizing systemic exposure and establishing robust biomarkers for target engagement. **Table 3** summarizes the major limitations and practical solution directions discussed above.

## 6 Summary and perspectives

As a nodal regulator linking redox balance to immune homeostasis and autoimmune-relevant pathways, Nrf2 exerts pleiotropic effects across heterogeneous arthritis subtypes by coordinating redox control, inflammatory signaling, and cell-death programs in the joint microenvironment. Instead of a uniform mechanism, Nrf2 integrates disease- and tissue-specific pathways: dampening osteoclast-driven bone erosion and curbing pathogenic FLS/macrophage activation in RA; intersecting with IL-23/IL-17, NF-κB, and STING axes in PsA/AS entheses and axial structures. Collectively, these findings support a unifying model where Nrf2 does not simply “add” antioxidant capacity, but selectively rewires pathological oxidative stress–inflammation circuitry and immunometabolism in distinct joint niches, and may also intersect with core immune-mediated processes—such as inflammatory antigen presentation, autoreactive effector amplification, and impaired resolution/regulatory programs—across distinct joint niches, laying a mechanistic basis for Nrf2-centered, microenvironment-oriented interventions in arthritis.

Technological innovations have expanded the translational landscape of Nrf2-targeted strategies. Genome-editing tools such as CRISPR/Cas9 enable precise modulation of Nrf2 and its regulators in preclinical arthritis models, facilitating causal dissection of pathway components and identification of druggable nodes (185). Integrative multi-omics, single-cell transcriptomics, and organoid models have begun to reveal tissue- and cell-type–specific Nrf2 programs across synovium, cartilage, subchondral bone, and immune compartments (186–188). Meanwhile, Nrf2 expression and activity emerge as candidate biomarkers reflecting local redox status and inflammatory tone: synovial Nrf2 activity correlates with inflammasome and cytokine signatures (87), while circulating Nrf2 levels link to RA inflammatory indices and composite clinical scores and dynamically change with treatment, supporting utility for disease monitoring and patient stratification (155). Therapeutically, ROS-responsive and joint-targeted nanocarriers, high-throughput screening platforms, and structure-guided design have accelerated the discovery of small-molecule and natural-product Nrf2 activators with improved potency and context-specific activation profiles (189–192).

Nevertheless, critical obstacles limit the clinical translation of Nrf2-based interventions in arthritis. Mechanistically, the crosstalk between Nrf2 and key pathways (NF-κB, NLRP3 inflammasomes, ferroptosis, immunometabolic reprogramming) remains incompletely mapped in human joint tissues, especially across disease stages and arthritis subtypes. Most functional evidence derives from rodent models, with limited robust validation in human synovial/cartilage samples, organoids, and *in vivo* imaging studies. Pharmacologically, most prototypical Nrf2 activators are electrophilic molecules with rapid metabolism, low oral bioavailability, and narrow therapeutic windows in humans (193, 194). To date, no Nrf2-targeted drug has shown unequivocal efficacy in arthritis clinical trials, and sustained systemic Nrf2 activation raises concerns of impaired host defense, tumorigenesis, and blunted physiological stress responses. These issues underscore the need for spatially and temporally controlled, joint-focused Nrf2 modulation over indiscriminate systemic activation.

Notably, conflicting observations on Nrf2’s role in bone remodeling across arthritis subtypes further complicate translation. In RA, Nrf2 consistently inhibits osteoclast overactivation and mitigates bone erosion, while emerging evidence suggests it may paradoxically promote osteogenic differentiation in AS by regulating Runx2 and OSX expression. This discrepancy likely reflects distinct microenvironmental ROS milieus: high ROS in RA synovium drives Nrf2 to prioritize antioxidant defense and inflammation suppression, while moderate ROS in entheseal tissues shifts its function toward controlling bone formation and structural remodeling. A second unresolved controversy involves Nrf2’s dual role in tumor biology and inflammation control. While Nrf2 activation benefits joint inflammation restraint, preclinical studies in oncology and synovial malignancies link chronic Nrf2 overexpression to enhanced chemoresistance and tumor progression, raising critical questions about the long-term safety of systemic, continuous Nrf2 activation—particularly in arthritis patients with elevated cancer risk.

Looking forward, addressing these unmet needs requires prioritizing five directions for rational development of Nrf2-centered therapies in arthritis. First, high-resolution mapping of Nrf2-dependent versus Nrf2-independent nodes in redox, inflammatory, and cell-death pathways is needed to identify context-specific targets and avoid mechanistic oversimplification. This effort should integrate single-cell and spatial multi-omics with perturbation experiments. Second, clinical translation should move beyond animal data toward systematic evaluation in human systems (synovial/cartilage organoids, explant cultures, and well-annotated patient cohorts). In addition, mechanism-based biomarkers and imaging readouts should be developed to monitor *in vivo* Nrf2 pathway modulation. Third, Nrf2 activators will likely work best in combination regimens: pairing redox-modulating agents with csDMARDs, bDMARDs, or JAK inhibitors may enable dose de-escalation, widen therapeutic windows, and address both inflammatory and oxidative components of joint damage. Fourth, inflammation-responsive or joint-targeted delivery platforms (enzyme-, pH-, or ROS-triggered nanocarriers; cartilage/synovium-homing conjugates) should be leveraged to concentrate Nrf2 activation in diseased joints while minimizing systemic exposure and long-term safety concerns. Fifth, clarify how Nrf2 shapes disease persistence and relapse by regulating immune memory programs—such as trained immunity in myeloid cells and the long-term maintenance of pathogenic versus regulatory lymphocyte states—which will be critical for developing durable Nrf2-centered strategies that prevent recurrence. Finally, cross-disciplinary collaboration across immunology, redox biology, materials science, and clinical rheumatology remains essential to align mechanistic insight, formulation design, and trial methodology. With these integrative efforts, Nrf2-based strategies may evolve from generic “antioxidant therapy” to precise, microenvironment-tailored interventions for diverse arthritis subtypes.
